# Evaluation of Single-Visit Post-endodontic Discomfort Using Three Different Rotary File Systems: An In Vivo Study

**DOI:** 10.7759/cureus.109655

**Published:** 2026-05-25

**Authors:** Sara Trivedy, Panna Mangat, Mampi Biswas, Akshata Gupta, Barkha Gupta, Roquiya Reyaz

**Affiliations:** 1 Department of Conservative Dentistry and Endodontics, Kalka Dental College, Meerut, IND

**Keywords:** nickel-titanium rotary instruments, postoperative pain, root canal therapy, single-visit endodontics, visual analog scale

## Abstract

Introduction

Postoperative pain following single-visit root canal treatment (RCT) remains a common clinical concern and is likely influenced by multiple factors, including instrumentation technique, apical debris extrusion, and mechanical stress on the periapical tissues. The aim of the present study was to evaluate and compare postoperative discomfort following single-visit RCT using three different rotary file systems.

Materials and methods

This prospective clinical observational study included 135 patients diagnosed with asymptomatic irreversible pulpitis of the posterior mandibular teeth. The participants were treated using one of three rotary systems: TruNatomy, GenEndo, and Jizai (n = 45 per group). All procedures were performed under standardized clinical conditions using a single operator. Postoperative pain was assessed using a visual analog scale (VAS) at 24 h, 48 h, 72 h, and 7 days. Statistical analyses were performed using IBM SPSS Statistics for Windows, Version 26 (Released 2018; IBM Corp., Armonk, NY, USA). Data were analyzed using the Friedman test for intragroup comparisons and the Kruskal-Wallis test with Mann-Whitney U post-hoc analysis for intergroup comparisons. Statistical significance was set at p < 0.05.

Results

All groups demonstrated a statistically significant reduction in VAS scores over time (p < 0.001). The TruNatomy group showed the lowest mean pain scores, decreasing from 1.84 ± 0.90 on Day 1 to 0.69 ± 0.76 on Day 7. In comparison, GenEndo showed a reduction from 4.07 ± 1.25 to 2.38 ± 0.89, and Jizai from 4.02 ± 1.27 to 2.60 ± 0.78. Intergroup analysis revealed significantly lower pain scores in the TruNatomy group than in the GenEndo and Jizai groups at all time points (p = 0.001), while no significant difference was observed between the GenEndo and Jizai groups (p > 0.05).

Conclusion

All rotary systems reduced postoperative pain over time. However, the TruNatomy group was associated with significantly lower postoperative pain scores compared to GenEndo and Jizai under the conditions of the present study. Its flexible and minimally invasive design may contribute to reduced periapical irritation and improved patient comfort. The selection of appropriate instrumentation systems can play an important role in minimizing postoperative discomfort in single-visit endodontic therapy.

## Introduction

Root canal treatment (RCT) is a fundamental procedure in endodontics aimed at eliminating infection and preserving natural dentition [1]. The success of RCT largely depends on effective chemo-mechanical preparation, which involves thorough cleaning, shaping, and disinfection of the root canal system [2]. Despite advancements in techniques and materials, postoperative discomfort remains a common clinical challenge, particularly in single-visit endodontic therapies. Pain following treatment can negatively influence patient perception, compliance, and overall treatment outcomes [3].

Post-endodontic pain is multifactorial in origin and is influenced by preoperative status, microbial factors, and operative procedures [4]. Among the intraoperative causes, apical extrusion of debris during instrumentation is considered a major contributor to periapical inflammation and subsequent discomfort [4]. With the introduction of nickel-titanium (NiTi) rotary systems, there has been a paradigm shift toward more efficient and minimally invasive canal preparation. These systems offer improved flexibility, better canal centering, and reduced procedural errors compared to manual instrumentation [5].

Contemporary rotary file systems, such as TruNatomy, GenEndo, and Jizai, incorporate advanced metallurgy and unique design features to enhance cutting efficiency while minimizing dentinal stress and debris extrusion [6-8]. TruNatomy (Dentsply Sirona, Ballaigues, Switzerland) is a heat-treated NiTi rotary system manufactured using proprietary post-manufacturing thermal treatment technology. It features a slim NiTi wire design, regressive taper, off-centered cross-section, and continuous rotary motion, which together enhance flexibility and preserve dentinal structure during canal preparation. GenEndo rotary files (Coltene/Whaledent AG, Altstätten, Switzerland) are conventional multi-file NiTi rotary instruments designed for sequential canal shaping using continuous rotational movement, with active cutting flutes intended to improve cutting efficiency and canal enlargement. Jizai (MANI Inc., Tochigi, Japan) is a heat-treated NiTi rotary system manufactured with controlled memory alloy technology and characterized by enhanced flexibility, variable taper design, and continuous rotational instrumentation, allowing improved canal centering and reduced canal transportation. Differences in alloy treatment, taper design, cross-sectional geometry, and kinematics among these systems may influence debris extrusion and postoperative pain outcomes [8].

Therefore, this study aimed to comparatively evaluate postoperative discomfort following single-visit RCT using three different rotary file systems. This study aimed to evaluate and compare the frequency and intensity of postoperative discomfort following single-visit root canal therapy using three different rotary file systems. The objectives of this study were to compare postoperative pain among the three rotary file systems, evaluate postoperative discomfort, and determine the rotary file system associated with the least postoperative discomfort, thereby identifying the most suitable system for enhanced patient comfort and clinical efficiency.

## Materials and methods

This prospective clinical observational study was conducted in the Department of Conservative Dentistry and Endodontics at the Kalka Dental College, Meerut, India, from May 2024 to June 2025. This study was performed in accordance with the principles of the Declaration of Helsinki. Ethical clearance was obtained from the Institutional Ethics Committee (KDC/LTR/2024/097; dated April 25, 2024), and written informed consent was obtained from all participants prior to treatment.

Sample size estimation

The sample size was determined a priori using GPower software (version 3.1; Heinrich Heine University Düsseldorf, Düsseldorf, Germany). Based on parameters derived from a previous study, an effect size of 0.30, α = 0.05, and statistical power of 80% were considered for an F-test (analysis of variance: fixed effects, three groups) [9]. The minimum required sample size was calculated as 120 patients (40 per group). To compensate for the potential dropout rate of approximately 10%, the final target sample size was increased to 135 patients, with 45 participants allocated to each group.

Study population and grouping

A total of 135 patients diagnosed with asymptomatic irreversible pulpitis involving mandibular posterior teeth were included in the study based on predefined inclusion and exclusion criteria. Patients aged between 18 and 60 years with restorable mandibular premolars and molars requiring primary RCT and exhibiting no clinical or radiographic evidence of periapical pathology were considered eligible. Only teeth with mature apices and clinically manageable canal anatomy were included. Patients with systemic diseases affecting pain perception or healing, those taking analgesics or antibiotics before treatment, pregnant or lactating women, those with teeth with acute apical abscess, swelling, sinus tract, retreatment cases, root resorption, calcified canals, open apices, severe canal curvature, periodontal involvement, or complex anatomical variations were excluded from the study to minimize confounding factors and ensure standardization among groups.

Mandibular posterior teeth were specifically selected because they commonly require endodontic treatment and generally present with relatively comparable root canal anatomy and occlusal loading conditions compared to anterior teeth. Additionally, these teeth are more frequently associated with postoperative discomfort due to their complex canal morphology and greater instrumentation demands. Restricting the study to mandibular posterior teeth helped reduce anatomical variability and improved standardization among study groups, thereby allowing a more reliable comparison of postoperative pain associated with the different rotary file systems.

The patients were treated using one of the three rotary file systems as part of routine clinical practice. The selection of the file system was based on operator preference and availability at the time of treatment, thereby reflecting real-world clinical conditions, rather than controlled allocation. The systems evaluated were TruNatomy (Dentsply Sirona, Ballaigues, Switzerland), GenEndo (Coltene/Whaledent AG, Altstätten, Switzerland), and Jizai (Mani Inc., Tochigi, Japan).

Clinical procedure

To minimize variability, all procedures were performed by a single experienced operator under standardized clinical conditions. Local anesthesia was administered, and rubber dam isolation was achieved in all cases. Access cavity preparation was performed using sterile burs. Glide path establishment was carried out using stainless steel K-files up to size #15. Working length was determined using an electronic apex locator and confirmed radiographically. Biomechanical preparation of the root canals was performed using the respective rotary file systems according to the manufacturer-recommended file sequence, taper, rotational speed, and torque settings. Apical patency was maintained using a #10 K-file between instrumentation steps, and final apical enlargement was standardized according to canal anatomy and the preparation protocol of the respective system.

Irrigation was standardized using 3% sodium hypochlorite and 17% ethylenediaminetetraacetic acid delivered with a 30-gauge side-vented irrigation needle placed approximately 1-2 mm short of the working length, followed by saline as the final rinse. A standardized volume of irrigant was used for all canals, and no additional irrigant activation method was employed. The canals were dried using sterile paper points and obturated in the same visit using gutta-percha and AH Plus resin-based sealer with the lateral compaction technique. Finally, the access cavity was restored using a temporary restorative material.

Outcome assessment

Postoperative pain was assessed using the visual analog scale (VAS). The VAS is a 10-point scale ranging from 0 to 10, where 0 indicates no pain, and 10 represents the worst possible pain. Pain intensity was categorized as follows: scores of 1-3, mild pain; 4-6, moderate pain; and 7-9, severe pain [10]. Patients were instructed on how to record their pain levels, and follow-up was conducted telephonically at 24 hours, 48 hours, 72 hours, and 7 days after treatment. Patients were advised to report any adverse events, and analgesics were prescribed only if necessary. Patients were instructed to prospectively note their pain scores at each predefined time interval using the VAS before the scheduled telephonic follow-up. Telephonic assessments were conducted using a standardized questioning format to minimize interviewer variability and maintain consistency in data collection. Due to the observational nature of the study, assessor blinding was not feasible. The collected data were compiled and subjected to statistical analysis to evaluate differences in postoperative discomfort among the three groups.

Statistical analysis

Statistical analyses were performed using IBM SPSS Statistics for Windows, Version 26 (Released 2018; IBM Corp., Armonk, NY, USA). Data normality was assessed using the Shapiro-Wilk test, which indicated a non-normal distribution of VAS scores. Descriptive statistics are expressed as mean ± standard deviation, median, and interquartile range. Intra-group comparisons across multiple time points were performed using the Friedman test, followed by Wilcoxon signed-rank post hoc tests with Bonferroni correction. Inter-group comparisons were conducted using the Kruskal-Wallis test, followed by the Mann-Whitney U test for pairwise analysis. Statistical significance was set at p < 0.05.

## Results

A total of 135 patients were included in the study and equally distributed into three groups (n = 45 each): TruNatomy, GenEndo, and Jizai. The demographic characteristics of the study population are shown in Table 1. Sex distribution did not differ significantly among the groups (p = 0.701). Similarly, the mean age of the participants was comparable across all groups, with no statistically significant difference (p = 0.973). These findings indicate that the groups were well matched and comparable at baseline.

**Table 1:** Demographic characteristics of study participants. Chi-square test was used for sex distribution, and one-way analysis of variance (ANOVA) was used for age. No significant difference between groups for either demographic variable (p > 0.05). Data are presented as frequency (%) or mean ± standard deviation (SD).

Parameter		TruNatomy (n = 45)	GenEndo (n = 45)	Jizai (n = 45)	Test value	p-value
Sex	Male, n (%)	25 (55.6%)	21 (46.7%)	23 (51.1%)	0.861	0.701
	Female, n (%)	20 (44.4%)	24 (53.3%)	22 (48.9%)		
Age (years)	Mean ± SD	35.2 ± 8.4	35.6 ± 8.6	35.3 ± 8.8	0.034	0.973

Intragroup comparisons of the postoperative pain scores at different time intervals are shown in Table 2. A statistically significant reduction in VAS scores over time was observed in all three groups (p < 0.001). In the TruNatomy group, the mean VAS scores decreased from 1.84 ± 0.90 on Day 1 to 0.69 ± 0.76 on Day 7. Similarly, the GenEndo group showed a reduction from 4.07 ± 1.25 to 2.38 ± 0.89, while the Jizai group demonstrated a decrease from 4.02 ± 1.27 to 2.60 ± 0.78 over the same period. Overall, all groups exhibited a progressive decline in pain scores, indicating resolution of postoperative discomfort with time.

**Table 2:** Intragroup comparison of postoperative pain scores at different time points with Friedman test. VAS: visual analog scale (0-10); Values reported as mean ± standard deviation (SD), median, and interquartile range (IQR). Data are presented for each group at four postoperative time points: Day 1, Day 2, Day 3, and Day 7. *p < 0.05 significant.

Group	Time point	Mean ± SD	Median	IQR (Q1-Q3)	χ²	p-value
TruNatomy	Day 1	1.84 ± 0.90	2.0	1.0 - 2.0	34.739	< 0.001*
	Day 2	1.56 ± 0.99	2.0	1.0 - 2.0		
	Day 3	0.98 ± 0.87	1.0	0.0 - 2.0		
	Day 7	0.69 ± 0.76	1.0	0.0 - 1.0		
GenEndo	Day 1	4.07 ± 1.25	4.0	3.0 - 5.0	44.736	< 0.001*
	Day 2	3.36 ± 1.23	3.0	3.0 - 4.0		
	Day 3	3.11 ± 0.98	3.0	3.0 - 4.0		
	Day 7	2.38 ± 0.89	2.0	2.0 - 3.0		
Jizai	Day 1	4.02 ± 1.27	4.0	3.0 - 5.0	35.390	< 0.001*
	Day 2	3.62 ± 1.13	3.0	3.0 - 4.0		
	Day 3	2.98 ± 1.22	3.0	2.0 - 4.0		
	Day 7	2.60 ± 0.78	3.0	2.0 - 3.0		

Post-hoc pairwise comparisons using the Wilcoxon signed-rank test are presented in Table 3. Significant differences were observed between most of the time intervals within each group. In all groups, comparisons between Days 1 and 3, and Days 1 and 7 were highly significant (p < 0.001). Significant reductions were also noted between Day 2 and subsequent time points. However, no statistically significant difference was observed between Days 1 and 2 in the TruNatomy group (p = 0.128) or between Days 3 and 7 in the Jizai group (p = 0.059). These findings suggested that the most pronounced reduction in pain occurred after the first 48 h.

**Table 3:** Post-hoc intragroup comparisons using the Wilcoxon signed-rank test. Adjusted significance level: p < 0.008; *Statistically significant.

Comparison	TruNatomy		GenEndo		Jizai	
	z-value	p-value	z-value	p-value	z-value	p-value
Day 1 vs Day 2	1.52	0.128	2.98	0.003*	2.14	0.032
Day 1 vs Day 3	4.21	< 0.001*	4.67	< 0.001*	4.23	< 0.001*
Day 1 vs Day 7	5.03	< 0.001*	5.44	< 0.001*	5.11	< 0.001*
Day 2 vs Day 3	3.45	0.001*	2.15	0.032	3.01	0.003*
Day 2 vs Day 7	4.12	< 0.001*	3.89	< 0.001*	4.02	< 0.001*
Day 3 vs Day 7	2.10	0.036	2.33	0.020	1.89	0.059

Intergroup comparisons of VAS scores at different time points are presented in Table 4. A statistically significant difference in postoperative pain among the three groups was observed at all evaluated time intervals (p < 0.001). Post-hoc analysis using the Mann-Whitney U test revealed that the TruNatomy group had significantly lower VAS scores than the GenEndo and Jizai groups at all time points (p = 0.001). However, no statistically significant difference was found between the GenEndo and Jizai groups (p > 0.05).

**Table 4:** Intergroup comparison of postoperative pain scores at different time points. The Kruskal-Wallis test was used for intergroup comparison. Post hoc analysis was performed using the Mann-Whitney U test with Bonferroni correction. Adjusted significance level: p < 0.017. *Statistically significant.

Time point	H statistic	p-value	TruNatomy vs GenEndo		TruNatomy vs Jizai		GenEndo vs Jizai	
			U statistics	p-value	U statistics	p-value	U statistics	p-value
Day 1	66.477	< 0.001*	138.0	0.001*	173.0	0.001*	1013.5	0.981
Day 2	58.954	< 0.001*	248.0	0.001*	177.5	0.001*	913.0	0.995
Day 3	66.901	< 0.001*	129.5	0.001*	193.0	0.001*	1121.0	0.998
Day 7	69.247	< 0.001*	190.0	0.001*	120.5	0.001*	877.5	0.725

## Discussion

Postoperative pain remains a significant concern in single-visit RCT and is largely influenced by mechanical, biological, and procedural factors [4]. The present study evaluated and compared postoperative discomfort following instrumentation using three contemporary NiTi rotary file systems. The findings demonstrated that all groups exhibited a significant reduction in pain over time; however, the TruNatomy system consistently showed significantly lower postoperative pain scores at all evaluated time points than the other two systems.

The progressive reduction in pain from Days 1 to 7 observed in all groups was consistent with the natural course of periapical tissue healing following endodontic therapy. This trend has been widely reported in previous studies, where postoperative pain is typically highest within the first 24-48 h and gradually subsides thereafter for up to one week due to the resolution of inflammation and reduction in microbial load [9,11]. The significant intragroup reduction in the VAS scores in the present study supports this well-established biological response.

The lower incidence of postoperative pain associated with TruNatomy can be attributed to its unique design and metallurgical properties. TruNatomy is a heat-treated NiTi system with a slim, regressive taper and enhanced flexibility, allowing it to conform more closely to the original canal anatomy while exerting minimal stress on the dentinal walls [12,13]. Previous studies have shown that instruments with higher flexibility and reduced taper tend to produce less apical extrusion of debris, which is the primary factor responsible for postoperative pain due to periapical inflammation [14]. Reddy et al. [15] demonstrated that TruNatomy files exhibit superior cyclic fatigue resistance compared to conventional endodontic file systems because of their advanced heat-treated NiTi alloys and enhanced flexibility. This improved mechanical performance may contribute to smoother canal preparation, with reduced stress and potentially lower postoperative discomfort. Additionally, reduced dentinal stress and minimal canal transportation associated with TruNatomy might have contributed to decreased irritation of the periapical tissues.

Supporting these findings, previous investigations have reported that TruNatomy exhibits lower dentinal stress and improved canal-centering ability compared to other rotary systems, thereby minimizing mechanical trauma during instrumentation [16]. Similarly, studies evaluating apical debris extrusion have demonstrated that although all rotary systems extrude some amount of debris, systems with a conservative taper and improved flexibility tend to reduce the extent of extrusion, thereby lowering postoperative discomfort [8,17].

In contrast, the GenEndo and Jizai systems demonstrated comparatively higher pain scores, although no statistically significant difference was observed. The lower postoperative pain scores observed with TruNatomy may potentially be related to its heat-treated alloy and minimally invasive design characteristics. GenEndo, a multi-file sequential system, may involve increased instrumentation steps, potentially leading to greater debris generation and apical extrusion. Additionally, a previous systematic review indicated that certain rotary systems may induce dentinal microcracks or increase stress within the canal, which can contribute to postoperative discomfort [18].

The findings related to Jizai are consistent with the literature, suggesting that although it exhibits good flexibility and shaping ability, its performance in terms of postoperative pain may be comparable to other systems rather than superior. Kyaw et al. [19] demonstrated that repetitive up-and-down movements during instrumentation significantly reduce torque and vertical force generation, with TruNatomy showing lower mechanical stress and surface wear than other systems. This reduction in operative stress may minimize periapical irritation and contribute to decreased postoperative pain. Kyaw et al. [20] reported that rotational speed significantly influences torque, force generation, and shaping ability, with optimized motion reducing stress on canal walls during instrumentation. Lower torque and controlled instrumentation dynamics may help to limit debris extrusion and mechanical trauma, thereby reducing postoperative discomfort. Jaggi et al. [21] demonstrated differences in debris extrusion and dentin preservation between systems using an in vitro model, highlighting the influence of file design and kinematics on mechanical outcomes. This discrepancy can be attributed to the difference in study design, as in vitro findings may not fully replicate clinical conditions in which host response, irrigation, and operator variability play a significant role in postoperative pain.

Although the differences observed among the groups were statistically significant, their clinical relevance should also be interpreted cautiously. In the present study, the TruNatomy group consistently demonstrated lower postoperative pain scores at all evaluated intervals, which may contribute to improved patient comfort and reduced anxiety following single-visit endodontic treatment. Even modest reductions in postoperative pain may enhance patient compliance and overall treatment acceptance in routine clinical practice. However, the magnitude of the observed differences should be interpreted within the limitations of the observational study design.

Clinical implications

From a clinical perspective, the findings of this study suggest that the choice of the rotary file system can influence postoperative patient comfort. The significantly lower pain scores associated with TruNatomy indicate that minimally invasive, flexible systems may be advantageous for reducing postoperative discomfort, particularly in single-visit endodontic therapy. Selecting instruments that minimize apical extrusion, preserve dentin, and reduce mechanical stress can enhance patient experience, improve compliance, and increase the overall treatment acceptance.

Limitations

Despite its strengths, the present study has certain limitations. As this was a prospective non-randomized observational study, allocation of the rotary file systems based on operator preference and instrument availability may have introduced selection bias and unmeasured confounding. Therefore, the observed differences among groups should be interpreted as associative rather than strictly causal. Although efforts were made to standardize clinical conditions by including only mandibular posterior teeth with asymptomatic irreversible pulpitis and by following uniform treatment protocols, tooth-specific anatomical variables such as canal curvature, root canal configuration, and number of canals were not independently stratified statistically and may have influenced postoperative pain outcomes. Pain assessment was based on patient-reported VAS scores and telephonic follow-up, which may be subject to subjective variability and recall bias. Additionally, the potential influence of analgesic consumption on postoperative pain scores was not analyzed separately. Since all procedures were performed by a single operator, operator-related variability was minimized; however, this may limit the generalizability of the findings. Future randomized controlled studies with larger sample sizes and stratification of anatomical and clinical variables are recommended to further validate these findings.

## Conclusions

Within the limitations of the present observational study, all three rotary file systems demonstrated a significant reduction in postoperative pain over time following single-visit RCT. Among the evaluated systems, the TruNatomy group was associated with consistently lower postoperative pain scores at all assessed time intervals compared with GenEndo and Jizai under the conditions of the study. Previous literature suggests that the heat-treated alloy, enhanced flexibility, and minimally invasive design characteristics of TruNatomy may contribute to reduced dentinal stress and lower apical debris extrusion; however, these parameters were not directly evaluated in the present study. Although GenEndo and Jizai demonstrated comparable outcomes, the findings suggest that minimally invasive rotary systems may be associated with improved postoperative patient comfort. Further randomized controlled studies are recommended to validate these observations.
